# Long-distance running: running for a long life?

**DOI:** 10.1007/s12471-014-0521-4

**Published:** 2014-02-13

**Authors:** E. E. van der Wall

**Affiliations:** Interuniversity Cardiology Institute of the Netherlands (ICIN) - Netherlands Heart Institute (NHI), P.O. Box 19258, 3501 DG Utrecht, the Netherlands

Physical exercise has been said to be healthy for the heart even in patients with various forms of cardiac disease [1–5]. A recent study published online in the European Heart Journal suggested that already being fit during the transition from the teenage years into adulthood may protect against heart disease decades later [6]. Men who had lower aerobic fitness levels when they were examined before entering mandatory military service at age 18 were more likely to have a myocardial infarction during a median follow-up of 34 years. Genetics likely play a large part in the aerobic fitness of teens but it could also be that men who are in good shape in their teens have adopted the sort of lifestyle that will keep them healthy later in life [7, 8]. What about the effects of extreme exercise, such as long-distance running [9], in particular marathons [10], or even longer distances than that, on health and physical well-being?

Running races longer than a marathon (42 km, 26.2 miles) have grown in popularity. In North America, 15,500 people finished such races in 1998 and more than 63,500 individuals in 2012, according to UltraRunning magazine. To that purpose, researchers from Stanford University School of Medicine and the University of California (USA) decided to investigate the health effects of extreme exercise. They assessed the physical health in a large population of ultramarathon runners to establish a baseline for the Ultrarunners Longitudinal Tracking (ULTRA) Study [11]. The results are based on an online survey of 1212 runners who had completed at least one race of at least 31 miles, of whom some had even performed 100-mile races. The findings were reported on Wednesday 8 January 2014 in the journal PLOS One [12]. The participants in the study were 18 to 81 years old, with an average of 42 years: mostly men (68 %) of Caucasian race. Their average running distance for the previous year was 3347 miles, being equal to an average of 64 miles a week.

In general, the study found that most of these runners are very healthy people, but most also do get injured in their activity. Important specific findings of the study were: 1) the ultramarathon runners had fewer carcinomas (4.5 %), heart disease (0.7 %) and diabetes (0.7 %) than the overall population-reported diseases, 2) the most prevalent findings were allergies and hay fever (25 %) and exercise-induced asthma (13 %), which are significantly higher than in the overall population occurring in only 7 to 8 % of people. The investigators attributed these phenomena to the amount of time runners spent outdoors; the asthma might result from the drying of the airways during exercise and the allergies from the increased exposure to airborne allergens, 3) a total of 77 % of the runners experienced exercise-related injuries and 64 % of the runners reported that an injury cost them training days, but those injuries rarely cost them time from daily activities. Runners lost an average of 2.2 days a year of work or school because of injury or illness versus 3.7 days for less active employed American citizens, 4) older and seasoned runners fared better than starting runners; younger and less experienced long-distance runners were more inclined to be injured, 5) people who ran more than 50 miles got more easily injured—stresses and strains and bruised toes; most of the injuries were to the legs, in particular knees and feet; 3.7 % of them were stress fractures, 6) finally, the study found that 5 % of ultrarunners were hospitalised after a competitive event in the past year due to dehydration, electrolyte disturbance or heat exhaustion.

The authors stated that this information is valuable in understanding potential benefits and risks from levels of exercise beyond the moderate amounts known to have health benefits. Assessing these runners over time will also help reveal how much exercise is optimal, how much recreational activity is appropriate and beneficial. The next step, a questionnaire to be sent early this year, will look at whether the runners have particular knowledge or adaptations to prevent injuries. The authors also hope to evaluate the psychological factors that motivate the ultramarathon runners.

To summarise, ultramarathon runners are generally healthier and take less sick time than the rest of the population. However, they tend to suffer from more knee pain, stress fractures, allergies and asthma than the general population. It therefore remains important for anyone planning to run marathons of any length to have a medical check-up first to make sure their heart and lungs are healthy enough for endurance exercise [13]. Lastly, one should always remember the old proverb: *exercise may increase the expectancy of life, but the extra years gained are spent exercising.*
